# Imatinib: A Breakthrough of Targeted Therapy in Cancer

**DOI:** 10.1155/2014/357027

**Published:** 2014-05-19

**Authors:** Nida Iqbal, Naveed Iqbal

**Affiliations:** ^1^Department of Medical Oncology, Dr. B. R. A. Institute Rotary Cancer Hospital, All India Institute of Medical Sciences, New Delhi 110029, India; ^2^Department of Anaesthesia and Intensive Care Unit, Indraprastha Apollo Hospital, New Delhi 110076, India

## Abstract

Deregulated protein tyrosine kinase activity is central to the pathogenesis of human cancers. Targeted therapy in the form of selective tyrosine kinase inhibitors (TKIs) has transformed the approach to management of various cancers and represents a therapeutic breakthrough. Imatinib was one of the first cancer therapies to show the potential for such targeted action. Imatinib, an oral targeted therapy, inhibits tyrosine kinases specifically BCR-ABL, c-KIT, and PDGFRA. Apart from its remarkable success in CML and GIST, Imatinib benefits various other tumors caused by Imatinib-specific abnormalities of PDGFR and c-KIT. Imatinib has also been proven to be effective in steroid-refractory chronic graft-versus-host disease because of its anti-PDGFR action. This paper is a comprehensive review of the role of Imatinib in oncology.

## 1. Introduction

Imatinib (also known as “Gleevec” or “Glivec”), a tyrosine kinase inhibitor, was called as “magical bullet,” when it revolutionized the treatment of chronic myeloid leukemia (CML) in 2001. Imatinib was invented in the late 1990s by biochemist Nicholas Lyndon then working for Ciba-Geigy (now Novartis), and its use to treat CML was driven by Brian Druker, an oncologist at the Dana-Farber Institute. The first clinical trial of Imatinib took place in 1998 and the drug received FDA approval in May 2001. Lyndon, Druker, and the other colleagues were awarded the Lasker-DeBakey Clinical Medical Research Award in 2009 for “converting a fatal cancer into a manageable condition” and the Japan Prize in 2012 for their part in “the development of a new therapeutic drug targeting cancer-specific molecules.” Encouraged by the success of Imatinib in treating CML patients, scientists explored its effect in other cancers and it was found to produce a similar miracle effect in other cancers where tyrosine kinases were overexpressed.

This review discusses the clinical implications of Imatinib in various cancers.

## 2. Clinical Pharmacology

Tyrosine kinases are important mediators of the signaling cascade, determining key roles in diverse biological processes like growth, differentiation, metabolism, and apoptosis in response to external and internal stimuli. Deregulation of protein kinase activity has been shown to play a central role in the pathogenesis of human cancers. Imatinib, a 2-phenyl amino pyrimidine derivative, is a tyrosine kinase inhibitor with activity against ABL, BCR-ABL, PDGFRA, and c-KIT. The active sites of tyrosine kinases each have a binding site for ATP. The enzymatic activity catalyzed by a tyrosine kinase is the transfer of the terminal phosphate from ATP to tyrosine residues on its substrates, a process known as protein tyrosine phosphorylation. Imatinib works by binding close to the ATP binding site, locking it in a closed or self-inhibited conformation, therefore inhibiting the enzyme activity of the protein semicompetitively [1]. This process ultimately results in “switching-off” the downstream signaling pathways that promote leukemogenesis. Imatinib also inhibits the ABL protein of noncancer cells, but cells normally have additional redundant tyrosine kinases which allow them to continue to function even if ABL tyrosine kinase is inhibited. Some tumor cells, however, have a dependence on BCR-ABL [2]. Inhibition of the BCR-ABL tyrosine kinase also stimulates its entry into the nucleus, where it is unable to perform any of its normal antiapoptotic functions [3]. Imatinib is well absorbed after oral administration with a bioavailability exceeding 90% [4]. It is extensively metabolized, principally by cytochrome P450 (CYP)3A4 and CYP3A5, and can competitively inhibit the metabolism of drugs that are CYP3A4 or CYP3A5 substrates. Interactions may occur between Imatinib and inhibitors or inducers of these enzymes, leading to changes in the plasma concentration of Imatinib as well as coadministered drugs [5]. Imatinib is generally well tolerated. Common side effects include fluid retention, headache, diarrhea, loss of appetite, weakness, nausea and vomiting, abdominal distention, edema, rash, dizziness, and muscle cramps. Serious side effects may include myelosuppression, heart failure, and liver function abnormalities [6].

## 3. Clinical Implications

### 3.1. Chronic Myeloid Leukemia

Chronic myeloid leukemia (CML) is characterized by the presence of a BCR-ABL fusion gene, which is the result of a reciprocal translocation between chromosomes 9 and 22 (Philadelphia (Ph) chromosome) [7]. BCR-ABL is the driving force of leukemogenesis in CML [8]. Being an inhibitor of BCR-ABL, the advent of Imatinib rapidly and dramatically modified the treatment of CML and led to important changes in management [9]. The initial landmark studies by Druker et al. showed high response rates to Imatinib in patients with advanced CML [10] and those pretreated with IFN-*α* [10, 11]. The IRIS study, a landmark study in CML, by O'Brien et al. compared Imatinib and the combination of interferon-alpha (INF-*α*) with cytarabine in a randomized trial in 1106 CP-CML patients [12]. Imatinib induced complete haematological response (CHR) in 95.3% patients and complete cytogenetic response (CCR) in 73.8% patients [12]. In addition, patients on Imatinib had a better quality of life [13]. On the basis of these results, Imatinib received FDA approval in December 2001. At 6-year follow-up of IRIS trial, Imatinib induced CHR in 98% of patients in chronic phase and CCR in 87% patients [14]. Response criteria in CML after treatment with Imatinib are given in Table 1.

The goal of therapy with Imatinib is achievement of major molecular response (MMR). Obtaining MMR was associated with significantly better long term remission-duration and progression-free survival (PFS). At 60-month follow-up, achievement of CCR and MMR by 12 months was associated with a PFS of 97% compared to 89% for patients with CCR but with less than MMR. Early molecular response predicted better outcome: progression of disease correlated with failure to achieve a 1-log reduction in transcript level by 3 months and a 2-log reduction by 6 months [14].

Despite a major clinical advance in the treatment of CML, Imatinib resistance has become a challenging problem. The existence of patients resistant to Imatinib was evident soon after the introduction of the drug into clinical practice. Initial responses were lower in patients with advanced-phase disease and responses tended to be transient in most responders with advanced-phase disease [15, 16]. Primary resistance is defined as an inability to achieve CHR at 3 months and MCR at 6 months. Primary resistance may be caused by differential drug metabolism and/or drug transport. Acquired resistance is defined as progression to advanced disease or loss of response with a 5–10-fold increase in BCR-ABL transcripts. Acquired resistance may be caused by mutations in the BCR-ABL kinase domain, amplification of the BCR-ABL fusion gene, overexpression of drug transporter genes, and overexpression of tyrosine kinases such as the SRC family kinases [17, 18]. Second-line treatment options include higher doses of Imatinib, a second-generation tyrosine kinase inhibitor (TKI), or allogeneic stem cell transplant (allo-SCT) [19, 20]. Phase III studies with second-generation TKIs like Nilotinib and Dasatinib have also shown superior efficacy to Imatinib in newly diagnosed CML, inducing faster and higher rates of CCRs and molecular responses. Therefore, both drugs are approved by the Food and Drug Administration to be used in patients with newly diagnosed CML in chronic phase (CML-CP) [21, 22].

### 3.2. Gastrointestinal Stromal Tumors

Gastrointestinal stromal tumors (GISTs) are mesenchymal neoplasms of the gastrointestinal (GI) tract accounting for <1% of primary gastrointestinal neoplasms. They are thought to arise from the interstitial cells of Cajal. GISTs are typically defined by the expression of c-KIT (CD117) in the tumor cells, as these activating KIT mutations are seen in 85–95% of GISTs. About 3–5% of the remainder of KIT-negative GISTs contain PDGFRA mutations [23, 24].

After CML, Imatinib dramatically altered both the management and prognosis for this rare disease [25]. Imatinib potently inhibits the tyrosine kinase activity of KIT. A number of clinical studies demonstrated the effectiveness of Imatinib in the treatment of unresectable or metastatic GIST [26–30]. These include studies examining the efficacy and tolerability of different doses of Imatinib (400 mg/day, 600 mg/day, or 800 mg/day) and different dosing regimens. In a phase III randomized trial involving 746 patients with advanced incurable GIST, 800 mg Imatinib was not found to be superior to 400 mg Imatinib as primary systemic therapy; no statistically significant differences in objective response rates, progression-free survival (PFS), or overall survival (OS) were observed [28]. However, in a phase II randomized trial examining dose selection in 946 patients with advanced GIST, patients whose tumors expressed an exon 9 KIT mutation, treated with a daily dose of 800 mg of Imatinib (versus 400 mg), experienced a significantly superior PFS (*P* = 0.0013) with a reduction of relative risk of 61% [31].

Surgery is the mainstay of curative treatment for patients with primary resectable GIST. However, a substantial proportion of GIST tumors have a high risk of recurrence and can be considered for adjuvant therapy [32, 33]. At least three phase III trials have evaluated the benefit of adjuvant Imatinib. In one randomized, double-blinded phase III trial, 713 patients who had undergone complete gross resection of a primary GIST measuring at least 3 cm and expressing KIT had been treated with 1-year Imatinib (400 mg daily) or placebo [34]. The 1-year relapse-free survival (RFS) rate was 98 versus 83 percent favoring Imatinib. The absolute benefit was greatest in those with high-risk disease (relapse rate 47 versus 19 percent for placebo and Imatinib, resp.); for moderate risk disease it was 14 versus 5 percent, respectively. No overall survival benefit was found. In another phase III trial, 908 patients with intermediate or high-risk GIST and including tumor rupture or intraoperative tumor spillage were randomly assigned to two years of Imatinib or observation alone [35]. At a median follow-up of 4.7 years, 5-year Imatinib-free survival (IFS) was 87 percent in the Imatinib arm compared to 84 percent in the control arm, 3-year RFS was 84 versus 66 percent, and 5-year overall survival was 100 versus 99 percent. The Scandinavian Sarcoma Group (SSG) XVIII trial compared 36 versus 12 months of adjuvant Imatinib (400 mg daily) in 400 patients with high-risk resected GIST [36]. At a median follow-up of 54 months, prolonged treatment was associated with a significant improvement in RFS and the primary endpoint (5-year RFS 66 versus 48 percent) as well as overall survival (92 versus 82 percent).

As in CML, resistance to Imatinib has proved to be a significant problem in GIST as all GIST patients treated with Imatinib for advanced disease will inevitably develop progressive disease. Primary resistance was seen in 12 percent of 934 patients in the randomized European trial exploring two different doses of Imatinib and was more likely in patients with lung but not liver metastases (41 percent) [37]. Several mechanisms of resistance to Imatinib in GIST have been explained. The mechanism of resistance most commonly observed is the emergence of new secondary mutations [38, 39]. Other identified mechanisms of acquired resistance have included amplification of KIT and pharmacokinetic resistance that may involve altered activity of drug transporters, induction of the cytochrome P450 (CYP)3A4 isoenzyme, and poor patient compliance [40, 41]. Dose-escalation of Imatinib or second-generation tyrosine kinase inhibitors (TKIs) may be considered in these settings [42–44].

### 3.3. Dermatofibrosarcoma Protuberans

Dermatofibrosarcoma protuberans (DFSP) is a rare soft tissue tumor accounting for approximately 1% of sarcomas with an indolent growth and a less than 5% probability of metastases [45]. DFSP is characterized by the presence of distinctive, reciprocal rearrangement of chromosomes 17 and 22. The rearrangement leads to the fusion of alpha chain type a (COL1A1) localized on 17q22 to the platelet-derived growth factor beta (PDGFB) localized on 22q13. The formation of COL1A1-PDGFB fusion gene results in the constitutional upregulation of PDGFB expression, leading to continuous autocrine activation of PDGF receptor B (PDGFRB) which is a key pathogenetic factor [46]. Based on the inhibitory effects of Imatinib on DFSP cell growth in various in vitro and in vivo studies [47], initial case reports showed the benefit of Imatinib in metastatic and locally advanced DFSP [48, 49]. In the largest retrospective series, 10 patients with locally advanced or metastatic DFSP received Imatinib 800 mg daily. All eight patients with locally advanced disease had karyotypic or fluorescence in situ hybridization (FISH) evidence of t(17;22), and all responded to therapy, two completely. The two patients with metastatic disease had more complex karyotypes. One who had the typical t(17;22) had a partial response that lasted seven months, while the second lacked the t(17;22) and did not respond [50]. It is not clear whether conventional DFSP tumors lacking t(17;22) respond as well to Imatinib. In neoadjuvant setting also, DFSP has been reported to be treated with Imatinib, with doses between 400 and 800 mg daily for a period ranging from 2 to 24 months (median, 4 months), producing an average tumor reduction of 50% (range: 19%–100%) after a median follow-up time of 24 months (range: 88 days to 72 months) [51–53]. Several questions remain regarding the mechanism of action of Imatinib and possible resistance to this targeted therapy in DFSP. However, Imatinib is currently the gold standard in the treatment of locally advanced or metastatic DFSP.

### 3.4. Philadelphia Chromosome-Positive Acute Lymphoblastic Leukemia

The Philadelphia chromosome (Ph) is a molecular abnormality present in approximately 30% of newly diagnosed cases of adult ALL. The occurrence of this disease subtype increases in an age-dependent manner and confers an unfavorable prognosis. Ph results from the translocation of chromosomes 9 and 22 producing a fusion gene, BCR-ABL [54]. Expression of BCR-ABL results in two different-sized proteins, p190 and p210. While p190 protein is exclusively expressed in Ph-positive (Ph+) ALL, p210 protein is predominant in chronic myelogenous leukemia (CML). TKI-based therapy has revolutionized treatment outcomes for this condition and has become the standard of care.

In a prospective, multicenter trial involving newly diagnosed Ph+ ALL patients, Imatinib was administered simultaneously or alternated with identical induction and consolidation chemotherapy [55]. Both treatment schedules allowed allo-SCT in a majority of the patients and produced tolerable adverse events, but concurrent Imatinib administration with chemotherapy produced greater anticancer effects.

The same group of researchers studied the long-term outcome of 335 patients on three different treatment schedules of Imatinib (600 mg/day) [56]. Patients in the first cohort received Imatinib between induction and first consolidation as well as after the first consolidation therapy. The second cohort was treated with Imatinib during the second half of induction chemotherapy and continued until allo-SCT. The third cohort started receiving Imatinib with induction chemotherapy and continued until allo-SCT. At 4-year evaluation, overall survival was found to be highest (50%) in patients in the third cohort. These findings suggest that Imatinib treatment should be started early with prolonged duration.

The single largest multinational, prospective study on two cohorts of Ph+ ALL patients showed similar results [57]. Patients in Cohort 1 received Imatinib following induction. Cohort 2 patients received Imatinib in the second phase of induction. Patients who were treated early with Imatinib responded better in terms of overall survival, event-free survival, and relapse-free survival. These findings again support the observation of treating Ph+ ALL patients earlier in therapy with Imatinib. So, Imatinib is approved as a first-line therapy for Ph+ ALL.

### 3.5. Hypereosinophilic Syndromes/Chronic Eosinophilic Leukemia

The hypereosinophilic syndromes (HES) are a rare group of disorders marked by the sustained overproduction of eosinophils, characterised by a blood eosinophil count >1.5 × 10^9^/L for at least 6 months without any recognizable cause and associated organ damage [58]. HES is a diagnosis of exclusion, once clonal eosinophilia (such as leukemia) and reactive eosinophilia (in response to infection, autoimmune disease, tropical eosinophilia, or cancer) have been ruled out [59]. Some HES patients are associated with a deletion in chromosome 4, which fuses the FIP1-like-1 gene (FIP1L1) to the PDGFRA gene, leading to FIP1L1-PDGFRA rearrangement. HES patients with a FIP1L1-PDGFRA rearrangement are now reclassified as chronic eosinophilic leukemia (CEL), as this gene has become a marker of disease clonality [60]. This fusion gene leads to constitutive activation of the PDGFRA TK [61]. Asymptomatic HES patients lacking evidence of organ damage are closely monitored, although there is no general consensus on how to treat them. The first-line treatment of HES has traditionally been prednisone, with a response rate of nearly 70%. However, relapses often occur, requiring the patient to seek second-line drug options, such as interferon-*α* or hydroxyurea [62]. The identification of FIP1L1-PDGFRA oncogene leads to the development of tyrosine kinase inhibitor as a therapeutic target. The frequency of this fusion gene is variable with various studies reporting a frequency between 3 and 56% [63, 64]. Imatinib induced complete hematological remission within three months in 100% of patients (15/15) [65]. Complete molecular remission, defined as a negative nested reverse transcriptase polymerase chain reaction (RT-PCR) for FIP1L1-PDGFRA fusion transcripts in peripheral blood, was seen within six months in 83% (10/12) of FIP1L1-PDGFRA-positive patients. In contrast, of the 14 patients lacking this marker, only three (21%) responded to Imatinib, while six (43%) achieved partial or complete clinical and hematological responses. FDA approved Imatinib as a first-line therapy in HES patients with FIP1L1-PDGFRA fusion protein.

### 3.6. Systemic Mastocytosis

Systemic mastocytosis (SM), a clonal neoplastic proliferation of mast cells, is defined by compact multifocal mast cell infiltrates in hematopoietic tissues with or without skin involvement [66]. SM is a heterogeneous spectrum of disorders; indolent to aggressive forms exist. Most patients with indolent SM (ISM) have a normal life expectancy. Advanced forms of mastocytosis include aggressive SM (ASM), mast cell leukemia (MCL), SM with an associated clonal hematological nonmast cell lineage disease (SM-AHNMD), and mast cell sarcoma (MSC) [66]. Mastocytosis is frequently associated with somatic gain-of-function point mutation with KIT. The most common somatic point mutation is the KITD816V, resulting from substitution of valine for aspartic acid at codon 816 within KIT exon 17 [67]. Imatinib was the first TKI evaluated in SM in vitro, but the results were disappointing [68, 69]. A study demonstrated an overall response (OR) in 5 out of 10 patients, and all responses were seen in patients negative for KITD816V mutation [70]. Imatinib was found to be effective in patients carrying KIT mutations other than those involving KITD816V in some case reports [71, 72]. Imatinib is the first and currently remains the only TKI that has been approved by the FDA for treatment of adult patients with ASM without the KITD816V mutation or with KIT mutational status unknown.

Apart from these cancers where Imatinib has already received FDA approval, various other cancers where Imatinib has provided dramatic responses include the following.

### 3.7. Aggressive Fibromatoses

Aggressive fibromatoses (desmoid tumors) (AF) are clonal fibroblastic proliferations characterized by infiltrative growths with a locally aggressive behaviour and no known metastatic potential [73]. Because of local invasiveness and high recurrence rates, they are associated with significant morbidity. Primary surgery with negative surgical margins is the most successful treatment modality for desmoid tumors. Radiation therapy may be used in recurrent disease or as primary therapy in unresectable patients [74]. The role of Imatinib in aggressive fibromatoses was explored by Mace and colleagues when they reported dramatic response to Imatinib in two patients with unresectable and progressive disease [75]. In a phase II Multicenter Sarcoma Alliance for Research through Collaboration (SARC) trial, Imatinib showed impressive response rates in 51 AF patients with or without previous treatment and locally advanced disease [76]. The PFS was 66% and 58% at 1 year and 3, respectively. Penel et al. treated 40 patients with unresectable and progressive symptomatic AF with Imatinib 400 mg daily for 1 year [77]. The 1- and 2-year PFS rates were 67% and 55%, respectively, while overall survival rate was 95%. None of the studies showed any significant correlation with the expression and/or mutations in Imatinib sensitive tyrosine kinase with outcome or response.

### 3.8. Malignant Melanoma

Malignant melanoma (MM) is a neoplasm of melanocytes. The incidence of malignant melanoma is increasing by 4% every year. The definitive treatment of cutaneous melanoma is surgery; medical management is reserved for adjuvant therapy of patients with advanced melanoma. Patients with melanomas arising from mucosal surfaces (e.g., sinuses, mouth, and vagina) or acral surfaces (e.g., non-hair-containing palms, soles, and nail beds) have very limited treatment options and survive less than 12 months in advanced disease. Mucosal and acral melanomas have different genetic alterations and biologic behavior compared with cutaneous melanomas. Recently, KIT-activating mutations were reported in 21% of mucosal melanomas, 11% of acral melanomas, and 16.7% of melanomas arising in chronically sun-damaged skin [78]. Additional cases showed increased KIT copy number or amplification. In a separate report, 15% of anal melanomas harbored a KIT mutation [79]. Due to a lack of effective therapies and discovery of KIT aberrations, studies on the use of Imatinib were initiated. A study by Carvajal et al. found that treatment with Imatinib in this subset of patients resulted in clinically significant response [80]. Recent studies also illustrated the effectiveness of Imatinib in patients with advanced melanoma harboring mutations or amplification of the KIT protooncogene [81, 82]. Of 50 patients with melanomas, 24 evaluable patients with KIT-mutant (*n* = 8), KIT-amplified melanoma (*n* = 11), or both (*n* = 5) were treated with Imatinib. Of these 24 patients, 7 achieved a partial response to therapy, with 5 patients' responses confirmed on subsequent imaging studies, for an overall confirmed response rate of 21% [81, 82]. Imatinib can be tried in MM having KIT aberrations.

### 3.9. AIDS-Related Kaposi's Sarcoma

Kaposi sarcoma (KS) is a spindle-cell tumor derived from endothelial cell lineage, associated with infection with human herpesvirus 8 (HHV-8). AIDS-related Kaposi sarcoma, unlike other forms of the disease, tends to have an aggressive clinical course. It is the most common presentation of Kaposi sarcoma [83]. Optimal control of HIV infection using HAART is an integral part of successful Kaposi sarcoma therapy [84]. However, patients with poor-risk Kaposi sarcoma rarely respond to HAART alone. Activation of c-KIT and platelet-derived growth factor (PDGF) receptors by autocrine and paracrine mechanisms follows Kaposi Sarcoma herpes virus (KSHV) infection of endothelial cells. In a recent phase II study, Imatinib demonstrated activity and was well tolerated in patients with AIDS-related Kaposi's sarcoma [85]. The study included 30 patients who received 400 mg Imatinib daily for up to 12 months. Overall, 10 patients (33%) achieved partial response and six patients (20%) demonstrated stable disease. Median time to response was 21 weeks and the median duration of response was 36 weeks. However, no correlation between c-KIT and PDGF mutations or any changes in the candidate cytokines with response was found. Imatinib may be considered as an alternative in some patients who progress on conventional therapy.

### 3.10. Chordoma

Chordomas are rare tumors that arise from embryonic notochordal remnants, comprising less than 1% of CNS tumors. Surgery is the preferred treatment; however, local relapses occur in >50% of cases. Metastases occur in at least 20% of patients [86].

A multicenter phase II clinical trial has confirmed the clinical efficacy of Imatinib in the treatment of chordoma [87]. Treatment with Imatinib was successful in stabilizing tumor growth (84%) or shrinking tumor size (16%) in a cohort of patients with progressing, advanced chordoma. The largest phase II study in patients with platelet-derived growth factor beta- (PDGFB-) positive advanced chordoma treated with Imatinib (800 mg daily) failed to elicit an overall tumour response defined by RECIST. However, at 6 months, 70% of patients remained stable during treatment, 64% showed a clinical benefit, and 18% showed some reduction in tumour size [88]. Due to lack of any therapy, Imatinib may be considered in advanced chordoma.

### 3.11. Recurrent Epithelial Ovarian Cancer

Most patients with epithelial ovarian cancer (EOC) relapse. The second-line therapy includes drugs, such as taxanes, topotecan, pegylated liposomal doxorubicin, and/or gemcitabine [89]. Platelet-derived growth factor (PDGF) and its receptor have been implicated in the early transformation and sustaining of tumor growth, their associated vascular endothelium, and signaling between tumor and stroma [90]. Various preclinical studies [91, 92] led to the use of Imatinib as a single agent for ovarian cancer, revealing some activity and some intolerance. In a phase II study, 14 patients with recurrent epithelial ovarian cancer (rEOC) were treated with Imatinib and weekly paclitaxel. The objective responses occurred in 4 patients and 5 of the 12 patients treated had a PFS of more than 6 months with minimal toxicities [93]. Though it was found to have some response, further studies on the role of Imatinib in rEOC are warranted.

### 3.12. Anaplastic Thyroid Cancer

Anaplastic carcinoma of the thyroid (ATC) is the most aggressive thyroid gland malignancy. A preclinical study of Imatinib showed efficacy in inhibiting growth of ATC cell lines [94]. Although the molecular target of this agent is not clearly defined, proposed mechanisms include inhibition of PDGF, KIT, and c-ABL. A single-institution study of Imatinib 400 mg twice daily orally in 11 patients with ATC was recently reported [95]. Of the eight evaluable patients, two had a PR, four had SD, and two had PD. Six-month PFS was 27%; 6-month survival was 46%. Frequent toxicities included lymphopenia, edema, anemia, and hyponatremia. Because of poor accrual, however, the trial was prematurely terminated.

### 3.13. Steroid-Refractory Chronic Graft-versus-Host Disease

Steroid-refractory chronic graft-versus-host disease (SR-cGVHD) is defined as progression despite treatment with prednisone 1 mg/kg per day for more than or equal to 2 weeks, or no improvement after 4 to 8 weeks of prednisone 0.5 mg/kg per day, or the inability to taper prednisone below 0.5 mg/kg per day [96]. Treatment for SR-cGVHD is challenging. Imatinib is a potent dual inhibitor of both transforming growth factor-b and platelet-derived growth factor receptor (PDGF-R) pathways, which are responsible for fibrosis and inflammation in cGVHD [97]. Imatinib also inhibits T-cell proliferation [98]. In a large phase II study, imatinib was tried in 40 patients with SR-Cgvhd [99]. After 6 months, out of 39 patients who received the drug, 14 had partial responses (PR), 4 minor responses (MR) with relevant steroid sparing (46%) according to Courriel criteria, and 20 more than or equal to PR (51.3%), as per the National Institutes of Health (NIH) criteria and NIH severity score changes. The best responses were seen in the lungs, gut, and skin (35%, 50%, and 32%, resp.). After a median follow-up of 40 months, 28 patients were alive, with a 3-year overall survival (OS) and event-free survival of 72% and 46%, respectively. There was a significant decrease in PDGF-R stimulatory activity in 7 responders, whereas it remained high in 4 nonresponders. So, Imatinib represents a valuable option for patients with SR-cGVHD.

## 4. Conclusions

The tyrosine kinase (TK) inhibitor, Imatinib, has revolutionized the therapy of malignancies that are addicted to one of its target kinases, c-ABL, c-KIT, and PDGFR. Currently, Imatinib is the standard of care in CML and GIST as it has dramatically changed the outlook of these diseases. Its use has extended to various other cancers and has achieved first-line position in cancers like Ph+ ALL, advanced dermatofibrosarcoma protuberans, hypereosinophilic syndrome, and systemic mastocytosis. Imatinib has also provided a valuable option in patients with SR-cGVHD who cannot access other treatments like extracorporeal photopheresis. No other targeted therapies have contributed so much to therapeutic armamentarium in oncology as Imatinib. Not only as therapy, Imatinib also acted as a tool for understanding the mechanisms of the diseases like CML and GIST. Various studies are ongoing to explore its benefits in other cancers also. The major drawback with Imatinib is development of resistance which is therapeutically challenging. Second- and third-generation TKIs have come up to overcome this resistance. Despite these limitations, Imatinib has contributed immensely to the field of oncology so that it should still be called a “wonder drug.”

## Figures and Tables

**Table 1:** Response criteria in CML.

Response	Criteria
Complete hematologic response (CHR)	Platelets <450 × 10^9^/L White cells <10 × 10^9^/L No circulating immature myeloid cells <5% basophils on differential No palpable splenomegaly

Minimal cytogenetic response (Minimal CR)	66–95% Ph+ cells*

Minor cytogenetic response (Minor CR)	36–65% Ph+ cells*

Partial cytogenetic response (PCR)	1–35% Ph+ cells*

Complete cytogenetic response (CCR)	No Ph+ cells*

Major molecular response (MMR)	BCR-ABL ≤0.10% (international scale)

Complete molecular response (CMR)	BCR-ABL transcripts nonquantifiable and nondetectable

*At least 20 metaphases analysed on conventional cytogenetics of bone marrow aspirate.
